# Advances in Testing Techniques for Digital Microfluidic Biochips

**DOI:** 10.3390/s17081719

**Published:** 2017-07-27

**Authors:** Vineeta Shukla, Fawnizu Azmadi Hussin, Nor Hisham Hamid, Noohul Basheer Zain Ali

**Affiliations:** 1 Electrical & Electronic Engineering Department, Centre for Intelligent Signal and Imaging Research (CISIR), Universiti Teknologi PETRONAS, 32610 Seri Iskandar, Perak, Malaysia; fawnizu@utp.edu.my (F.A.H.); hishmid@utp.edu.my (N.H.H.); 2 Waham Technologies, Subang Jaya, 47600 Selangor Darul Ehsan, Malaysia; noohulina@hotmail.com

**Keywords:** digital microfluidics, testing, reliability, MEDA, droplet, biochips

## Abstract

With the advancement of digital microfluidics technology, applications such as on-chip DNA analysis, point of care diagnosis and automated drug discovery are common nowadays. The use of Digital Microfluidics Biochips (DMFBs) in disease assessment and recognition of target molecules had become popular during the past few years. The reliability of these DMFBs is crucial when they are used in various medical applications. Errors found in these biochips are mainly due to the defects developed during droplet manipulation, chip degradation and inaccuracies in the bio-assay experiments. The recently proposed Micro-electrode-dot Array (MEDA)-based DMFBs involve both fluidic and electronic domains in the micro-electrode cell. Thus, the testing techniques for these biochips should be revised in order to ensure proper functionality. This paper describes recent advances in the testing technologies for digital microfluidics biochips, which would serve as a useful platform for developing revised/new testing techniques for MEDA-based biochips. Therefore, the relevancy of these techniques with respect to testing of MEDA-based biochips is analyzed in order to exploit the full potential of these biochips.

## 1. Introduction

The mixed-technology based microsystem has an emerging category known as microfluidics. Microfluidics or Lab-on-a-chip (LOC) involves the processing of small volume of fluids (typically in the range of 10^−6^ to 10^−18^ L) in small channels that have dimensions in the range of micrometers [1,2]. These microfluidic biochips are widely employed in various biomedical applications including parallel immunoassays, drug discovery and point of care disease diagnosis, monitoring infectious disease and to detect pathogens. Most of the commercially available biochips are continuous flow microfluidic biochips in which the flow of liquid is controlled by micro-pumps, micro-valves, electro-kinetics and electro-osmosis [3,4]. However, permanently-etched channels and complex external assistance restrict the feasibility and versatility of these biochips [3]. Thus, the focus is shifted from Continuous Flow Based Microfluidics to Digital Microfluidics Based Biochips (DMFBs). These devices have the capability of handling very small volumes of liquids. Results can be provided in a prompt manner and test circuits can be easily operated [2]. The computing functions and sensing modules in the digital microfluidic biochips can potentially enhance the microfluidic technology [1].

**Figure 1:**
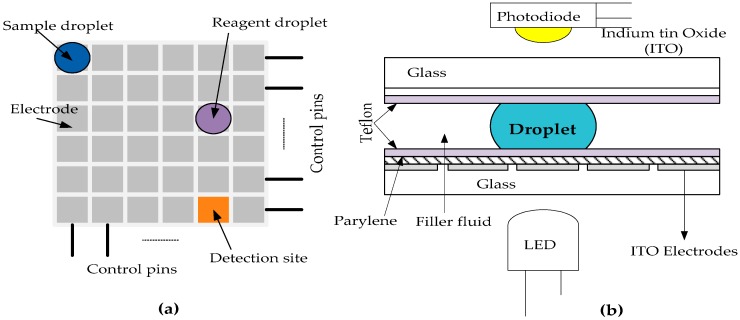
The schematic view of a DMFB (**a**) Top view of a digital microfluidic biochip system, (**b**) Cross-section view of the digital microfluidic system showing the sandwiched droplet between the parallel plates.

Generally, a DMFB comprises of electrodes arranged in the form of arrays in two-dimensional (2D) surface as shown in Figure 1a,b. It also consists of other exterior components such as droplet detector and droplet dispensing port. The sample and reagents consist of very small volumes of discrete droplets which are controlled by the electrodes based on the principle of Electro-Wetting-On Dielectric (EWOD) [1,2]. These droplets are moved from one position to another in the 2-D array with the help of time-varying voltage pulses applied to the controlled electrodes [2,5].

In recent years, the system level integration and complexity of DMFBs have increased rapidly due to increasing requirements of multiple and concurrent bioassay operations [1]. The recently proposed Micro-electrode-Dot-Array (MEDA) architecture-based digital microfluidic biochips have the potential to integrate and execute multiple and complex operations concurrently [6]. Like conventional DMFBs, the principle of Electro-Wetting-On Dielectric (EWOD) is utilized in MEDA to move the droplet from one micro-electrode to the adjacent micro-electrode [6,7]. The well-known CMOS fabrication technology is used to fabricate MEDA-based biochips [6]. Additionally, some advanced operations provided by this architecture such as diagonal movement of droplet and channel-based routing make it suitable to be used in any environment without any directional constraints [6,8].

The difference between conventional DMFBs and MEDA-based biochips lies in the architecture [8]. MEDA-based biochips are based on the sea-of-micro-electrodes having similar basic units called micro-electrode cells (MCs) [6]. The MEDA-based biochips contain micro-electrodes that are ten times smaller (typically ~100 μm) than the electrodes conventionally used in digital microfluidics [6]. The micro-electrode cells are connected together using a scan-chain structure owing to the high integration possible in MEDA-based biochips. The micro-electrodes are activated dynamically to form droplets of varying shape and size [8]. As compared to the conventional DMFB where the basic unit is a physical electrode, each MC in MEDA-based biochip has a physical micro-electrode (fluidic domain) and a control circuitry (electronic domain) beneath it [9].

The MEDA-based biochips provide several advantages over conventional digital microfluidic biochips. MEDA architecture allows dynamic grouping of the micro-electrodes to form droplets of arbitrary shape; hence, its operation is different from those of the conventional digital microfluidics biochips [9]. Additionally, conventional DMFBs suffer from various limitations including constraints on droplet size, incapability to change the volume of droplets in a fine-grained manner and lack of integrated sensing systems for the detection of droplets in real-time situations [9,10]. The MEDA-based biochips are able to form a longer contact line in order to control the droplets [6]. Furthermore, the diagonal splitting in MEDA-based biochips can be done more successfully than the conventional splitting as a more effective contact line is provided by the surrounding micro-electrodes. It also allows fast mixing procedure by creating multilaminates [7,8]. Thus, MEDA-based DMFBs could provide a good platform for exploring potential scalability and reconfigurability in DMFBs [6].

However, the increased complexity of DMFBs, the underlying mixed energy domain and the use of new materials have led to numerous potential defects in DMFBs upon manufacturing and during the in-field operations [11]. The effects of these manufacturing defects may become more apparent during the execution of bioassays [12]. Additionally, parametric variations such as electrode size and oxide thickness would affect the biochip performance during the bioassay operations. Some physical defects like particle contamination may happen during the operations of digital microfluidic biochips [13]. Thus the reliability of these biochips becomes a critical factor when they are used in various safety critical applications [13]. Various testing techniques including built-in-self test (BIST), error recovery, and design for testability have been proposed in the literature for the testing of DMFBs [11,13,14]. However, the presence of mixed domains in MEDA-based biochips, the complexity of their architecture due to the density of large number of basic units (micro-electrodes) and the complex control circuitry requires additional test procedures [10]. Therefore, the testing techniques employed for conventional DMFBs should be modified before they are applied in MEDA-based biochips [10].

In this paper, we provide an overview of testing techniques used for conventional digital microfluidics and their applicability for MEDA-based digital microfluidic biochips. A comparison of these testing techniques is presented based on offline (manufacturing testing) and online testing (in-field testing), test completion time, type of sensing circuit at sink and size of microfluidic array used. Additionally, an overview of MEDA-based biochip is presented, focusing on its architecture and the recently proposed test techniques for this architecture. Fault models for the MEDA-based biochips are presented to gain better understanding of the defect types in these DMFBs.

The paper is organized as follows: Section 2 explains the testing procedures that are commonly used in conventional DMFBs. Section 3 describes different types of testing techniques used for conventional DMFBs. Section 4 provides an overview of MEDA-based biochip, its architecture and presents the fault models for MEDA-based biochips. Section 5 concludes the current work and offers some suggestions for future work.

## 2. Testing of Digital Microfluidic Biochips

When a DMFB operation does not meet the desired specification, it indicates that there is a fault in the biochip. Typically, electrical methods are used to detect faults in microfluidic biochips. The faults occurred in DMFBs may be due to imperfections produced during the manufacturing process or degradation of the electrode during field operations. These faults in DMFBs are either catastrophic (hard) or parametric (soft) in nature [1,11]. Catastrophic faults cause overall failure of the digital microfluidic biochips whereas parametric faults lead to degradation in DMFB performance. Some of the possible catastrophic defects include dielectric breakdown, insulator degradation, short-circuit between the electrodes, etc. Deviations in geometric parameter and variations in droplet viscosity and filler medium are some of the examples of parametric defects. The growing use of digital microfluidics in various medical applications has triggered the need for developing effective test approaches to guarantee its dependability [1,11,13,15].

**Figure 2:**
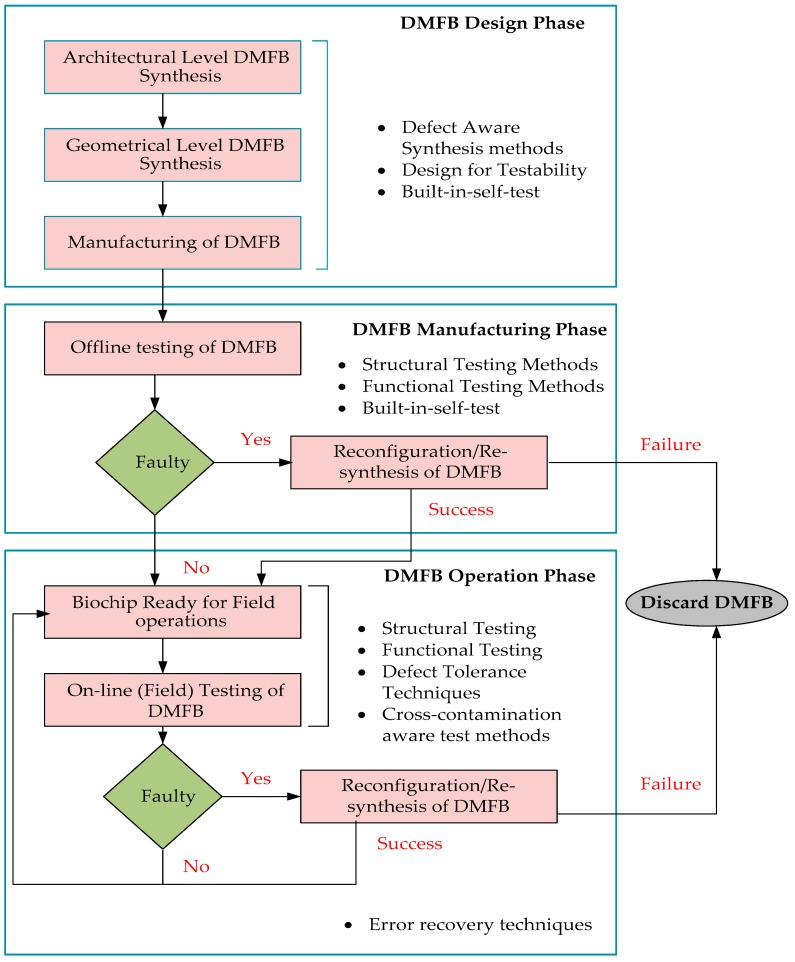
Design flow and test of Digital Microfluidic Biochips.

Generally, testing of digital microfluidic is a process which includes movement of test droplet in the microfluidic array following a predefined routing path. The first breakthrough in designing digital microfluidics was reported by Fan et al. [16] where an electro-wetting-on dielectric (EWOD)-based digital microfluidic chip was fabricated having a 9 × 9 grid array and where basic fluidic operations (e.g., transportation, cutting and merging of droplets) can be performed. Fault modeling of lab-on-chips involving flowFETs has further guided the way for DMFB testing [17]. The testing of digital microfluidic biochips follows a generic flow of procedures performed during the design stage, the manufacturing process and the assay operation [13]. This flow is shown in Figure 2. The testing of DMFBs starts from the beginning of design phase where defect-aware synthesis method, design for testability and built–in-self test can be applied. After the DMFB manufacturing process, it is tested for any manufacturing defects. If any defect exists in the DMFB, reconfiguration techniques are then adopted.

The main criteria for the testing approach can be broadly divided into two categories: offline testing and online testing [13]. In the offline testing approach, the biochip is tested after the manufacturing process for any defect before it is used in the field. The biochip is discarded if it is deemed faulty due to manufacturing defects. On the other hand, in the online testing approach, the testing of biochip is done concurrently with the bio-assay. The online testing is applied to the DMFBs so that the defects in the biochips can be identified during the bio-assay operations. If any defect is found in DMFB during the online bioassay operation, error recovery techniques and reconfiguration techniques are applied to ensure proper performance of bioassay [15]. Many parameters have been taken into account during the testing of biochips such as testing time, placement of modules, routing plan, fluidic constraints, etc. [12]. If the testing techniques fail to deliver the desired performance after manufacturing or during in-field operations, then the biochip is discarded.

In the following section, the testing techniques for conventional DMFBs will be presented in a more elaborate manner. These testing techniques have shown to test the conventional DMFBs in an efficient manner. We have analyzed these testing techniques in order of their effectiveness throughout the development of various digital microfluidic biochips.

## 3. Testing Techniques for Conventional Digital Microfluidic Biochips (DMFBs)

Recently, a considerable amount of work has been done in the field of digital microfluidic biochip testing [12,13,17]. The most general form of testing of a DMFB is structural testing where a test droplet moves from source to sink electrode within the two-dimensional array of electrodes on a pre-defined VLSI routing path. If the droplet appears at the sink after the completion of the routing path, the DMFB is said to be fault-free [18]. The subsequent section describes various testing approaches that have been proposed in the literature so far.

### 3.1. Structural Testing Techniques

The testing of digital microfluidic biochips can be initiated by moving a test droplet in a predefined path from source to sink. This approach is called structural testing [18]. An Integer Linear Programming (ILP) approach was firstly used for structural testing [18,19,20]. A unified mechanism for defect detection was proposed by Su et al. [18]. This concurrent testing approach was used to test catastrophic as well as parametric faults in DMFBs. The test droplets were moved on a predefined routing path of the DMF array which is based on the ILP scheduling approach. The method was validated by performing real-time bioassay on an array of 15 × 15 size. An obstacle-avoiding routing algorithm based on ILP was presented by Chang et al. [19]. The algorithm used for ILP was less complex and it was able to achieve high routability. The ILP-based routing method was also used by Mitra et al. [20] for offline washing of biochips to remove contamination in regular and irregular biochip geometries. A unified detection method was presented to detect catastrophic and parametric defects in the DMFBs [21]. Monte-Carlo simulation was done to study the effects of fluidics and physical parameters on DMFBs. However, as the ILP problem is treated as NP-hard in nature, heuristic solutions were required to solve the problem. Thus, Monte-Carlo and real time search algorithms were proposed [22].

**Figure 3:**
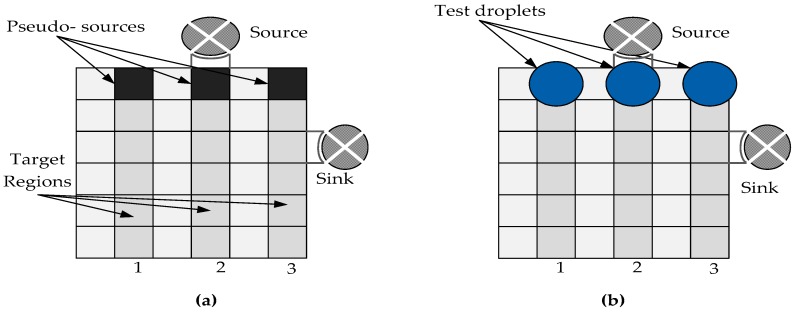
Parallel scan like testing in DMFB (**a**) Test droplets are dispensed from the source to the start electrodes known as pseudo-sources. (**b**) An example of test droplets traversing parallel in the columns of electrodes array [27].

However, due to the increased complexity in the optimization problem, the ILP-based methods are computationally costly when the size of microfluidic array increases. Thus, graph-based testing methodology has been presented to overcome the computational cost of ILP method [23,24,25,26]. Using graph-based methodology, a biochip was modeled as an undirected graph and it was then eulerized. A flow path obtained based on the Euler theorem allowed the test droplet to move easily on the array. A graph-based testing methodology applicable for online and offline applications was proposed by Su et al. [23]. Their testing methodology focused on the detection of catastrophic faults including electrode-shorts faults. In this approach, however, the test application time increases if no edges are available during online testing. This is because the test droplet has to wait at the current cell (electrode) until a free edge is accessible. Additionally, the proposed approach was based on single fault assumption. Mitra et al. [24,25] proposed an improved eulerization test routing technique based on graph model. Here, an optimal eulerization was abstracted in terms of the classical Chinese postman problem. The classical Hierholzer’s algorithm was then used to recognize the Euler tour. The Hierholzer’s algorithm depends on the cycle decomposition method. The test time was significantly reduced and this algorithm was able to be used in test-based route planning. A multi-droplet detection graph based technique was proposed by Majumdar et al. [26] for detecting single fault in DMFBs. These testing droplets were traversed in parallel to perform multi-droplet detection. The test droplets underwent a special movement pattern, i.e., Right-up-Right-Down (RURD). These test droplets scanned the middle cells and edges of the Euler graph. The scanning of boundary cells and edges was done in an anti-clockwise manner. The Euler-based method had shown improvement in terms of fault detection as compared to the existing approaches of structural testing. However, the completion time of Euler path test increased as the size of array increased. Therefore, it was difficult for a single droplet to move from source to sink consisting of very large arrays that involve thousands of electrodes. To tackle this issue, parallel testing approach was presented [27,28,29,30,31]. Parallel testing was introduced by Xu et al. [27] in which parallel test droplets were routed on the electrode arrays. Parallel droplet paths can be used in both online and offline testing. For parallel testing methodology, each array was assigned a testing droplet and a target region as shown in Figure 3. In this testing approach, the start electrodes were treated as pseudo-sources as shown in Figure 3a and the multiple test droplets were moved in parallel from these pseudo sources (Figure 3b). These test droplets traversed parallel to the target regions from where the droplets merged and finally routed to the sink reservoir for further analyzing.

This method is similar to the single droplet scan-like testing. The droplets move in parallel; thus, this method is called parallel scan-like testing. To detect the testing droplets, an enhanced capacitive sensing circuit was employed at the sink reservoir to detect the pulse sequence of multiple test droplets. The testing time significantly reduced to approximately 75% of that required by the Euler test method due to the reduced complexity of fault diagnosis from O(N2) to O(N). Similarly, an integrated testing and diagnosis method was proposed by Davids et al. [28] to locate single and multiple defects with high fault coverage without flooding. In this methodology, structural testing was used for the outer loop of microfluidic array and parallel testing was used for the inner arrays using multiple droplets. Capacitive sensing circuit was employed for the droplet detection at the sink electrode. The diagnosis process involved calculating the number of time steps required to attain the test droplet from the suspicious set.

A structural testing approach that combines both fault detection and fault recovery was proposed by Chen et al. [29] in order to reduce the reliability problem in DMFBs. The microfluidic array was partitioned and the combined binary search and parallel scanning approach was applied to test electrodes in the array.

**Figure 4:**
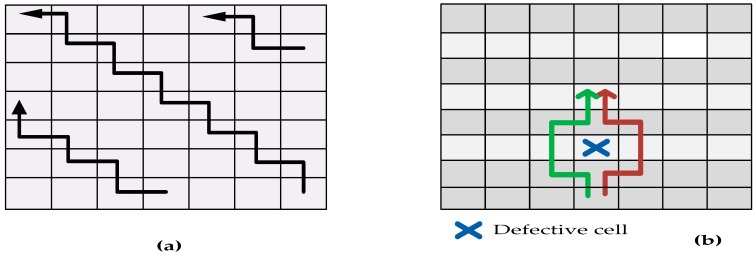
Structural testing (**a**) Diagonal scan-like test to detect undetectable faults; (**b**) Local detouring for high error rate [29].

Diagonal scan-like test (as shown in Figure 4a) was also used to identify undetectable faults which could not be diagnosed using parallel scan-like testing approach. These undetectable defect locations occur when there are multiple defects in the DMFB. Thus, diagonal testing was used where multiple test droplets traversed the array in diagonal manner. Additionally, a binary partitioning was performed repeatedly in order to match any one of the following conditions: (1) there is no undetectable cell in the entire sub-array or (2) there is at least one defect in each row or column. However, the binary portioning becomes impractical when the array is too large and the error rate is high. In this case, local detouring was used as shown in Figure 4b. The local detouring is used whenever there is a defect around the defective cell. Although this local detouring decreases the chance of having undetectable cell, it increases the run-time of error detection algorithms due to the slow movement of test droplet. This problem was tackled by utilizing a more efficient algorithm by selecting the Initial Traversing Depth (ITD).

A parallel testing approach based on a look-ahead strategy using multiple droplets was proposed by Roy et al. [30,31]. Their main aim was to minimize the test application time and optimize the consumption of test resources. In this look ahead strategy, selection of a predetermined path in the layout (which is defect-free) was done in such a way that the test droplet can be moved safely and the test time can be optimized. Additionally, Hightower’s line search-based routing algorithm was used to find the shortest path. A fault diagnosis method based on distributed dispensing and scheduling of multiple test droplets in a time synchronized manner was reported by Mukherjee et al. [32] to avoid routing conflicts in DMFBs (better fault detection). Parallel multiple assays on a restricted sized chip were performed by Dhal et al. [33]. As reported, the probability of locating faults was lower if faults occur at locations far away from the sink and the source. Additionally, some faults could not be detected by parallel testing.

Test time minimization has been the major aim of structural testing in DMFBs. Several works have been done in this area to minimize the test completion time as some assays are time constrained [33,34,35]. The approach proposed by Das et al. [34] was able to reduce the testing time by partitioning the DMFB array into clusters and applying the routing path to each cluster individually. A new pipelined scan-like testing method proposed by Li et al. [35] had managed to minimize the test completion time. The approach was based on the selection of appropriate parameters such as voltage and frequency of actuating the electrodes. The distribution of electric field and its effect on dielectric degradation on the electrodes can then be examined by these parameters. Additionally, pin-constrained chips were used to solve the problem of large number of pins involved in actuating the electrodes (hence minimizing test time). In these biochips, several modules can be identified by the same pin number. The reliabilities of pin-constrained chip designs was investigated by Huang et al. [36] who proposed effective algorithm based on general models to overcome the reliability issues in Pin-constrained digital microfluidic biochips (PDMFBs).

### 3.2. Functional Test Methods

**Figure 5:**
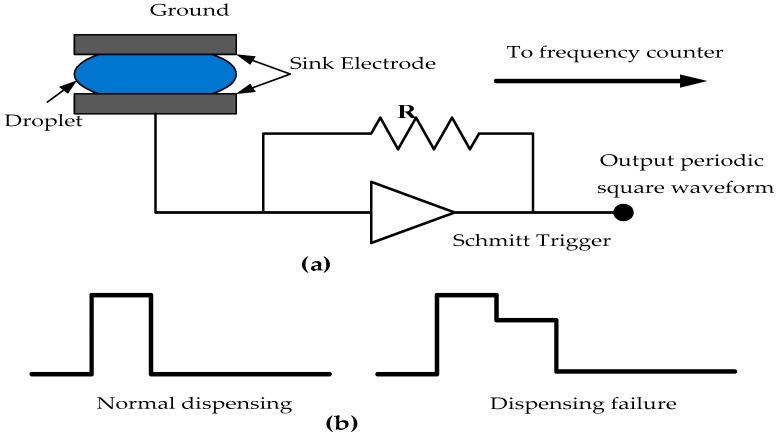
Functional testing (**a**) capacitive sensing circuit with droplet in the sink electrode (**b**) test readouts from the sensing circuit for the defect detection in DMFBs [38].

Functional test methods have been proposed to overcome the limitations of structural test methods as these methods are inadequate for testing fluidic operations performed on the array such as mixing/splitting of droplets and dispensing of droplet from the source [37,38,39]. Structural testing approaches dealt with the presence/absence of test droplet at the sink by moving the test droplets across the microfluidic arrays. Since, mixing/splitting and droplet dispensing operations may suffer from volume variation during in-field operations which causes parametric variations, additional test methods were required to test the volume variation of droplet in these functional modules of DMFBs [37,38]. Additionally, the structural test methods do not cover the testing of non-reconfigurable components of biochip system such as capacitive sensing circuits. The occurrence of any defect would lead to parametric as well as catastrophic failure during the bioassay operation. Thus, functional testing must be performed to validate the reliabilities of these biochip components. In order to ensure proper operations of functional units such as mixing, splitting and capacitive sensing various approaches were proposed [37,38]. A capacitive sensing based dispensing was used by Xu et al. [38] to detect the droplet dispensing failures as shown in Figure 5. The droplet volume variation due to any defect was detected at the sink electrode with the help of an oscillator circuit (Figure 5a). This oscillator circuit outputs the capacitance of droplet in the form of waveform as can be seen in Figure 5b. In the defect-free case, full waveform is expected and the dispensing failure is reflected as a deviated waveform from the oscillator output.

In this approach, two threshold values of pulse amplitude obtained from the calibration of sensing circuit were used to identify two abnormal droplets. Thus, the sensitivity (normal, oversensitive or insensitive) of the capacitive sensing circuit was determined by the amplitude of positive pulse. Additionally, functional testing for mixing operation was done by testing the “merge and route operations” within the target electrode clusters. In order to achieve robust assays execution, Mitra et al. [39] proposed accelerated functional testing. Fewer possible steps were used to detect major defects so that the test time could be reduced and the electrode degradation resulting from the application of extreme actuation voltage could be minimized. An optimal bidirectional routing test and an accelerated test for mixing/splitting that require very few droplet manipulation steps and lesser execution time was presented in their work. The approach by Shih et al. [40] implemented a sensing and feedback control system for the monitoring of droplet movements in such a way that if any failure is observed, the application of additional driving voltages could help the droplet to complete the desired task. This sensing system was evaluated using liquids such as water and methanol. This sensing control system was simple and inexpensive.

The main disadvantage of functional testing is the increased assay completion time due to the detection of core defects and malfunctions. One of the reasons behind this is that fewer unit cells are available for the assay operations in fault detection and fault location and less parallelism is used in the synthesis process during assay execution. Furthermore, the complexities involved in capacitive sensing circuits and the associated test analysis pattern in the functional testing make it unfeasible for field operations.

### 3.3. Defect Tolerance Techniques

Defect tolerance techniques have been proposed so that a DMFB is capable of performing assay operations even when there are defects on the biochip [41]. Generally in DMFBs, defect tolerance is realized by including redundant elements in the system to replace the faulty elements by using reconfiguration techniques. Another substitute for defect tolerance has been based on graceful degradation. In this technique, the elements in the system are treated in a uniform manner without being assigned as spare elements. When any defect occurs, a subsystem is chosen from the faulty system to perform the desired operation functionality but with a gracefully-degraded level of performance in terms of longer operation time.

A popular and efficient defect tolerance technique for DMFBs was proposed by Su et al. [42]. The dynamic re-configurability inherited in DMFBs was adopted by the proposed approach to bypass the faulty electrodes in the array. Three reconfiguration techniques, i.e., local, partial and full reconfigurations with different fault tolerance methodologies were presented. This methodology ensured that the biomedical assays will be able to perform well even in defective biochips using these reconfiguration techniques. The proposed approach was evaluated by real-time biomedical assays. The reconfiguration approach ensures a longer lifetime of biochip and a higher production yield. However, the reconfiguration cost increased due to the utilization of spare cells located at the boundary row/column. In order to overcome this, an interstitial approach was incorporated [43]. The spare cells were placed in the interstitial sites of the DMFB array. These spare cells would replace any faulty cells which were situated adjacent to it. Thus, defect tolerance was achieved effectively by using the local reconfiguration techniques.

Placement of microfluidic modules plays an important role to ease the re-configurability of fault tolerance. Thus, Su et al. [44] presented a module placement technique based on simulated annealing. Due to NP-completeness of placement problem, module placement technique based on simulated annealing was used so that the problem can be solved in a computationally efficient manner. Later, for large microfluidic arrays, Su et al. [42] proposed a defect tolerance technique based on graceful degradation of electrodes and dynamic reconfigurations of DMFBs. A scalable tile-based DMFB architecture was introduced for large bio-assay applications. Arrays of reconfigurable tiles in this architecture were used to perform basic microfluidic operations.

Apart from reconfiguration techniques, Zhao et al. [44] presented an Automated Test Pattern Generation (ATPG) method for non-regular layout of digital microfluidic biochips. This ATPG can be fully utilized on the non-regular layout where reconfiguration techniques are not fully utilized. Due to the automation of test stimulus design and test resource selection, the test time was minimized. To compact the test patterns, an ILP-based model was presented while preserving the preferred fault coverage. For the detection of catastrophic defect, the electrode was treated as a “buffer”. The input of this buffer was connected to the previous electrode. The test droplet moved from the previous electrode through the buffer to the output electrode which was connected at the buffer output. Dijkstra’s algorithm was also presented to solve the problem of selecting the reservoir and determining the minimum routing path. Additionally, the redundant test patterns were deleted by the process called test pattern-compaction.

The defect tolerance techniques have proved to be efficient when the error occurs during in-field operations of DMFBs. However, these techniques lead to an increase in the area overhead of the biochips due to the addition of spare elements. Additionally, the bio-assay operation time also increases in case of degraded performance which is not suitable for the bio-assay applications which are time specific.

### 3.4. Built-in-Self-Test (BIST)

**Figure 6:**
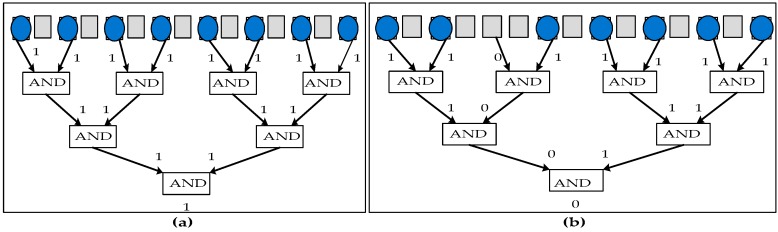
Microfluidic compactor used for parallel scan like testing with gray boxes representing electrodes, blue circles represent the test droplets and the arrows represent the output of AND logic gate being fed to the input of another AND logic gate: (**a**) compactor output = “1” which represents the test droplet at the sink electrode and the DMFB is defect free (**b**) compactor output = “0” which represents the absence of test droplet at the sink electrode and the DMFB is faulty [47].

Due to the complexity of analyzing test outcomes in terms of pulse sequences, sensitive nature of capacitive sensors and inaccuracies in sensor calibrations, the Built-in-Self-Test (BIST) method has been proposed for biochips [45,46,47]. However, as stated earlier, the use of capacitive sensing circuits and examination of the pulse sequence of the output makes the previously proposed methods impractical in field operations. Therefore, microfluidics compactors based on droplet AND gates were utilized in BIST testing methodology [47]. For the AND gate implementation, logic values “0” and “1” were referred as absence and presence of liquid droplet, respectively. This approach guaranteed low area overhead and it was able to operate bio-assays due to the dynamic reconfiguration of compactors used. All test droplets were compressed by the compactor used in this technique into a “signature “droplet. This “signature” droplet can then be detected at the sink by a photo-detector composed of photo-diode and LED as shown in Figure 6a,b, avoiding the requirement of using capacitive sensing circuits. The use of a compactor reduces the number of sources and sinks to only one; thus, the costs of fabrication and chip packaging can be reduced. The use of compactor ensures zero aliasing, thus providing complete fault coverage of single as well as multiple electrodes as it does not mask any error in the microfluidic array. A very small array has been used for the verification of the compactor.

The extended work by Zhao et al. [47] presented AND, OR and NOT microfluidics logic gates. The BIST architecture was applied to the pin-constrained digital microfluidics biochips. This architecture was effectively evaluated by using a multiplexed bioassay protocol. However, in pin-constrained DMFB, as the input pins are shared between the electrodes, the problem of electrode interference may appear due to unintentional droplet manipulations. The BIST architecture made up of AND gates could not be realized properly in the pin-constrained chip. This is because of the conflicts that arise due to the steps needed by BIST architecture for fluidic and pin-constrained droplet operations. The input pin may be required by the BIST architecture (test operation) and the fluidic operations (bio-assay operations) at the same time. Thus, a BIST-aware pin-constrained design was presented in their work. Their design approach supported the target bioassay as well as the BIST architecture which include the test techniques and the test results from the droplet compaction. The proposed BIST architecture was also applied to parallel scan-like testing and functional testing. In the case of parallel scan-like testing, a microfluidic compactor was placed to compress the multiple droplets coming from the parallel arrays of biochips. This microfluidic compactor consist of a combination of OR and NOT gates for the functional testing. However, one of the disadvantages of using compactor was that it did not cover some of the untestable defects as mentioned in the previous parallel testing approaches. These approaches focused on the defect detection in the DMFBs but not on the location of defects in the DMFB array.

To overcome the above limitations, a fault diagnosis approach was proposed by Zhao et al. [46] for the detection of defective cells in single and multiple rows or columns in microfluidic arrays. An output compactor based on microfluidics exclusive-OR gates was used in this approach which compresses 2n distinct test outcomes to an n-droplet signature. Thus, the need of using capacitive sensing circuit to analyze the outcome was eliminated. The proposed diagnosis approach was evaluated by analyzing the probability of misdiagnosis which was done by calculating the compression ratio as defined below:

(1) $$\mathrm{Compression}\;\mathrm{ratio}=\frac{\mathrm{Compressed}\;\mathrm{Size}\;\mathrm{of}\;\mathrm{bits}}{\mathrm{Uncompressed}\;\mathrm{Size}\;\mathrm{of}\;\mathrm{bits}}$$

where “Uncompressed Size” is the number of bits of the test vector result before the compaction process and “Compressed Size” is the number of bits in the code word after the compaction process.

### 3.5. Design for Testability

The percentage of functional components or electrodes that can be tested efficiently on a synthesized chip is defined as testability [48]. Design-for-testability (DFT) is achieved by incorporating test procedures into the fluidic manipulation steps in the target bio-assay protocol. A testability approach was developed by Xu et al. [48] for digital microfluidics biochips. In this DFT method, a test design was integrated into the basic fluidic operations of a particular assay procedure. A pin-constrained irregular layout of biochips was considered where the unused electrodes were removed from the electrode arrays to decrease the production cost. However, there existed a tradeoff in the form of additional constraints that these droplet manipulation steps must satisfy. Due to these constraints, the test techniques can become completely inefficient or can only affect a small portion of the biochip. This ultimately reduces the testability of the biochip. Thus, an efficient DFT solution was proposed by the authors to tackle the abovementioned problem. This approach guaranteed lesser completion time for assay operations. In this approach, the testability was divided into two categories namely structural testability and functional testability. The percentage of electrodes that can be tested during structural testing defines structural testability. Similarly, the percentage of functional components (mixing/splitting/dispensing modules) that can be tested using functional testing defines functional testability. Thus, high testability indicated high fault tolerance and increased flexibility for the design. However, in the pin-constrained design of biochips, functional testing could not be applied directly to the functional modules due to problem of electrode interference [48]. Thus, the issue was resolved by applying the test-friendly pin assignment in the early stage of chip design. A broadcast addressing based pin-constrained design was also developed for the proposed test-aware design method.

Further, a test methodology was proposed by Sheikh et al. [49] to monitor the working status of electrodes in DMFBs by classifying the electrode cells into weak, faulty and fault-free cells. By using 180 nm technology, a measurement circuit was built in order to classify the cells into these three categories. The design-for-testability approaches promised to increase the yield of DMFBs. However; there is also a necessity to verify the correctness of on-chip fluidic operations. These approaches are inefficient in monitoring the volume of droplets and sample concentration in the product obtained, which can lead to erroneous assay outcomes when there is a defect in the biochip. Thus error recovery approaches have been proposed in the literature to overcome this limitation.

### 3.6. Error Recovery

The test approaches mentioned in the previous subsections did not considered the problem of recovering from the fluidic errors that can occur during the on-chip bio-assay operations references. Further, the fluidic manipulations on the DMFBs were carried out without any feedback and the error could only be detected once the whole bio-assay operation is completed. Thus, in the case of any defect, the whole bio-assay operations must be repeated. This ultimately increased the wastage of samples and reagents as well as bio-assay execution time [50,51]. Thus, there was a need to monitor the intermediate results which could be accomplished by inserting a feedback mechanism in the DMFB system during the bio-assay execution so that only part of bio-assay is repeated where the errors are detected.

Various error recovery mechanisms have been proposed for the DMFBs [50,51,52,53,54,55,56,57]. Error recovery can also be incorporated at the synthesis level. A synthesis approach integrating the error recovery mechanism in the DMFBs scheme was proposed by Zhao et al. [50]. The location of fluidic checkpoints was determined by using error propagation method during biochip synthesis. The proposed method was evaluated using real life bio-assays and the completion time was reduced to approximately 30% of that required by the biochip with a control path during the bio-assay implementation. Here the control path was monitored using the control mechanism for the digital microfluidic biochip. The intermediate product droplet was then checked for any defect at the checkpoint where an on-chip photo-detector or a capacitive sensing circuit was situated.

However, checkpoint observation and rollback retrieval mechanism led to increased assay completion time. Thus, Luo et al. [51] proposed a “physical aware” system reconfiguration technique. This technique exploited the sensor data at the checkpoints so that the chip can be configured dynamically. A resynthesis technique was then applied to update the sequence of actuation of electrodes. As a result, new schedules, module placements and routing paths could be generated for the droplets with minimum response time. The proposed method had alleviated the need to perform the whole assay again (if defect was found) by applying a transformative “cyberphysical” approach. In this cyber physical system, re-synthesis process starts when an error is detected at the checkpoint. The biochip was configured dynamically by synchronizing the physical aware control software and the biochip. This allowed the sensor data at the immediate checkpoint to be used as feedback. Additionally, a sensing system based on Charge–Coupled Device (CCD) and optical detector was also proposed in their work.

Further, Luo et al. [52] proposed an integrated hardware rapid error recovery approach (based on an error dictionary). Because of the limited memory available in the hardware (micro-controller), two compaction techniques were used to store the error dictionary. The proposed approach had shown less impact on the response time. Also, the approach was able to be implemented easily on the experimental setup and required lesser memory space in storing the error dictionary. The proposed approach overcomes the shortcomings of previous error recovery approaches which required online resynthesis (if error occurs) and inability of cyberphysical system to be used in flash chemistry. Later, a hardware assisted method was presented Luo et al. [52] that can be implemented in real-time on FPGA. The data compaction technique was used to store the error dictionary in the limited memory space of FPGA. The proposed approach was evaluated using four laboratory protocols and it gives lower response time. Likewise, this approach also utilized a simple experimental setup and a small memory space in the form of FPGA for the dictionary.

An experimental demonstration of coordination between control software and hardware (biochip and sensors) was presented by Hu et al. [54]. Here, the errors were detected by using the capacitive sensors and the test outcome was presented by control hardware. The software-based recovery was done using dynamic reconfiguration. These hardware interfaces were implemented using off-shelf micro-controller. Shift register were used for electrode addressing and FPGA was used for the implementation of frequency divider. Jaress et al. [56] had proposed a compiler and a runtime monitoring system for cyber-physical DMFBs to enable fast dynamic fault recovery. A cyberphysical control algorithm that detect hard and soft faults rectifies dynamically while running the biomedical assay on the biochip was presented. The approach was shown to be scalable and it was able to run efficiently in practical applications; thus, the incurred performance overhead when a hard or soft fault occurs online was limited. Error recovery has proven to be useful for detection of defects in the on-line bioassay operations. However, it shows some limitations for bio-assay operations which are time dependent. The feedback inserted in the microfluidic array increases the portion of assay completion time which is needed to be considered.

### 3.7. Defect-Aware Synthesis Methods

Apart from ensuring defect-tolerance and error recovery at online operation of bio-assays, the synthesis level of DMFBs has also incorporated defect-tolerance feature [58,59,60,61,62,63]. The main advantage of this feature is that the DMFBs are capable of performing assay operations by adjusting various parameters such as module placement and routing path in case of any defect. Firstly, a simulated annealing based module placement technique was proposed by Su et al. [58] for the DMFBs. In the placement procedure, the fault tolerance was considered so that the microfluidic module can be relocated to other places when defects are found on the electrode. A unified synthesis approach that combines architectural synthesis with defect aware physical design was presented by Xu et al. [59]. Here, the main design criterion was defect-tolerance which allows simultaneous processing of architectural level and defect-tolerant physical designs. The defect-tolerant synthesis was applied on the protein and PCR bio-assays, thus leading to the marginally larger array area and increased time/cost as compared to the defect-oblivious approaches due to the direct consequence of using the reconfiguration techniques.

A droplet routing approach was presented by Zhang et al. [61] for a fault-tolerant DMFB. The proposed technique featured fluid handling operations simultaneously and sensors integrated with the biochip were used for run-time diagnosis. Error recovery was incorporated in this work. The error recovery operation started as soon as fault was detected in the circuit. A synthesis approach was proposed by Alister et al. [64] for fault tolerant application-specific biochip architectures. By utilizing this approach, the redundant electrodes were used to help designers to tolerate catastrophic defects and to increase the yield of biochips. Using this synthesis approach, the allocation and placement of modules and their interconnections could be decided. The optimization problem of placement was solved by applying Simulated Annealing (SA) method. Furthermore, a physical level synthesis flow was proposed by Liao et al. [65]. This approach took into account the variation, defects and contamination of DMFBs. The routing technique for variation, contamination and defect was based on the maze routing integrated to the existing placement technique. The defect-aware synthesis methods provide a promising direction in the testing of digital microfluidic biochips. However, most of the defect-aware synthesis approaches are application specific which restrict their use when a biochip is fabricated for general purpose applications.

### 3.8. Cross-Contamination Aware Methods and Wash Droplets

During the bioassay operations, the leftovers of residue liquid (from different molecules of samples and reagents) might create contamination problem in digital microfluidic biochips. To overcome this issue, the concept of wash droplets has been proposed in the literature. The wash droplets clean the contaminated surfaces of the electrodes arrays after/before the bio-assay operations so that it does not interfere with other subsequent bio-assay operations. Several works have been done in this area [20,66,67,68,69,70,71,72,73,74]. 

A contamination aware droplet routing algorithm was proposed by Chiang et al. [67] in order to schedule the wash droplets without the necessity of using extra electrodes. Thus, the execution time did not increase and it helped to improve the reliability and the fault tolerance of DMFB. Additionally, Minimum Cost Circulation (MCC) was utilized so that multiple wash droplets can be used. Similarly, a routing aware placement technique was presented by Roy et al. [68] for the digital microfluidics biochips with a particular prescheduled arrangement of module. The main objective of this placement algorithm was to improve the routing of the droplet by utilizing intelligent collision avoidance and optimized stalling and detour. Due to this reason, the resource utilization and the latest arrival time could be improved.

A concurrent routing scheme was proposed by Pan et al. [72] for multiple washing droplets. The approach ensured that the droplets work with minimum cross contamination. Additionally, a droplet schedule operation for the removal of residue from the contamination site was also proposed. A droplet routing flow was highlighted by Wang et al. [74]. The author studied realistic issues including the constraint of finite washing capacity and the routing conflicts happened between the functional and washing droplets. In order to minimize the washing capacity consumption, functional droplet paths were considered during washing routing. An effective A* algorithm was utilized to find satisfactory paths with minimized crossings. When timing constraints were considered, an efficient compaction algorithm was proposed so that the routing of functional and washing droplets could be scheduled properly.

The remaining residue in the bioassay may mix with the droplets of subsequent bio-assays, which can lead to defective biochips in subsequent bio-assay operations. Hence, the cleaning of pathways by wash droplets is required. An efficient route plan for the wash droplet could minimize the wash time and/or electrode actuation. A wash operation synchronization method was presented by Zhao et al. [66] in order to clean the residues via handling of the wash droplets. The synchronization of wash droplets routing with bioassay droplet routing scheme was done by controlling the order of arrival of droplets at the site of cross-contamination. This resulted in minimization of routing time and it was shown to be effective for chips with tight area constraints. An efficient approach of residue removal in DMFBs was presented by Mitra et al. [20]. The authors formulated the routing problem in terms of graph eulerization and capacitated Chinese postman problem. The use of multiple washing droplets improved the washing time. The use of wash droplets on the surface of electrode increased the reliability of bio-assay operations, hence the DMFBs. However, the presence of washing droplets in the concurrent bio-assay processes increases the risk of defects caused by them such as “stuck wash droplets” in their path, which hinders the routing path of bio-assay operations and can result in erroneous assay outcomes.

We have discussed various testing approaches proposed in the literature so far. It can be seen from the above testing techniques that the conventional DMFBs suffer from many defects starting from the design phase to the operation phase. Thus, various testing techniques have been applied at different abstraction levels of DMFBs. A brief summary of possible defects in DMFBs and the associated testing techniques is presented in Table 1 [37]. It can be seen from the table that most of the defects in DMFB can be tested using structural and functional testing. However, these techniques are impractical if the testing time is prolonged in the case where the bio-assay operations are time-based.

Thus, more advanced approaches such as error recovery, design for testability, defect-aware synthesis methods can be used to overcome the limitations of structural and functional testing. However, error recovery approaches, design-for-testability and defect-aware synthesis methods are useful in error detection during the bio-assay operations. As discussed earlier, the architectural difference in conventional DMFBs and MEDA-based biochips requires additional measures to be taken for the testing of MEDA-based biochips. The MEDA-based biochips include defects in both fluidic and electronic domains. The testing techniques applied for conventional DMFBs only focused on the fluidic domain (i.e., electrode surface) of DMFBs. Thus, the testing techniques for conventional DMFBs cannot be applied directly to the MEDA-based biochips [9,10]. The detailed explanation of architecture of MEDA-based biochips and related testing issues will be explained in the upcoming section.

## 4. Testing of Micro-Electrode-Dot Array (MEDA)-Based DMFBs

**Figure 7:**
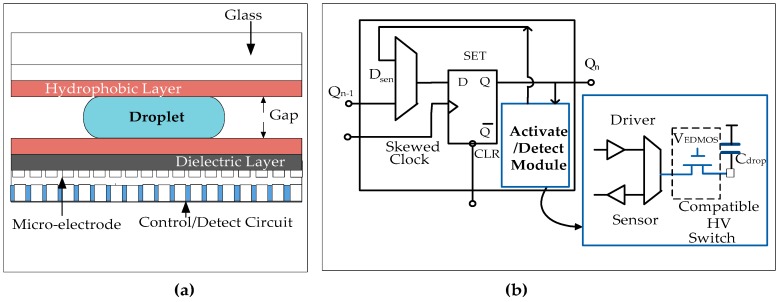
(**a**) MEDA architecture of biochip consisting of physical micro-electrode and the control circuitry underneath. (**b**) The detailed architecture of control and detect circuit beneath the micro-electrodes [10].

MEDA-based biochips possess scalability, portability and configurability in its architecture. As explained earlier, the conventional DMFBs and MEDA-based biochips differ in their architecture [75]. The routing algorithms that are applied to conventional DMFBs are not partially applicable to MEDA-based biochips as this architecture follows an additional movement called diagonal movement of droplets [9]. The basic architecture of MEDA-based biochips consists of a fluidic domain (physical micro-electrode) and an electronic domain (control/detect circuit). The fluidic domain has two parallel plates as shown in Figure 7a. There is an electronic activation circuitry beneath the physical micro-electrode called control/detect circuit as shown in Figure 7b.

The bottom plate consists of patterned micro-electrodes which are individually connected to the electronic part (activation circuitry) that lies beneath these physical micro-electrodes. The activation circuitries are fabricated in a low-voltage CMOS process. In order to achieve CMOS compatibility, an extended drain MOSFET (EDMOS) called high voltage switch is used under a 3V power supply in order to increase the avalanche breakdown voltage to 25V (Figure 7b) [9,10]. Therefore, the droplets can be actuated easily using the high breakdown voltage (25 V) of the EDMOS switch. In the fluidic domain, the upper electrode is connected to the ground. A hydrophobic layer (typically Teflon or Cytop) is also applied on both surfaces of electrodes in order to decrease the wettability of the surface and to produce a high contact angle (Figure 7a). The bottom micro-electrodes are coated with a dielectric material (typically Parylene C or SiO_2_) in order to increase the value of applied voltage and to avoid short-circuiting. Due to this dielectric layer, the potential difference is distributed around the system when it is charged. A droplet of finite volume is sandwiched between these two plates [75]. This droplet sits on multiple micro-electrodes in MEDA-based structure. The shape and size of the droplet are changed by dynamically activating the micro-electrodes [8]. The basic operations of MEDA-based biochips include transportation, mixing and splitting of droplets during the bio-assay and detection of the product at sink. As the use of MEDA-based biochips is increasing in biomedical applications, the reliabilities of these biochips become critical. Thus, advanced testing techniques are required for MEDA-based biochips [9,10]. New testing techniques involve defining fault models for MEDA-based biochips. Thus, the existing fault models are modified in terms of MEDA architecture. The upcoming subsections also describe some of the recent testing methodologies used for MEDA-based biochips.

### Fault Models for MEDA-Based Digital Microfluidic Biochips

In DMFBs, physical defects at some level of abstraction are represented by fault models. All fault models are applicable to MEDA-based biochips when only a single micro-electrode is considered during the test operation. When multiple micro-electrodes are involved in a bio-assay, the above-mentioned testing techniques for the biochips must be modified to enhance the testing efficiency. The development of testing methodology for MEDA-based biochips requires the development of associated fault models. Thus, we have developed fault models for MEDA-based biochips to cater for cases involving multiple micro-electrodes and defects in one or more micro-electrodes. Table 2 shows the fault models and observable errors for MEDA-based biochips where N represents the number of micro-electrodes [76].

As seen in Table 2, the observable error for one micro-electrode (N = 1) is the same as the conventional DMFBs. However, when more than one micro-electrodes (N > 1) are actuated together to form a droplet, the error in any one of the micro-electrodes is not catastrophic in nature but behaves parametric in nature [13,76]. Some of the defect types which cause different observable error in MEDA based biochips are described below:

- *Dielectric Breakdown*: This defect happens due to the application of high voltage on the micro-electrode. Thus, a short is created between the droplet and the physical micro-electrode. When this defect is considered in conventional digital microfluidics involving one droplet per electrode, droplet electrolysis will take place and transportation of the droplet is ceased. However, in the case of MEDA-based biochips, since the droplet consists of many micro-electrodes, defect occurred in any micro-electrode will lead to the localized electrolysis in the micro-electrode. This would result in a reduction in the number of resultant droplet and consequently affects the biomedical assay operation due to droplet slow-down. This defect only affects the fluidic domain of the biochip; the electronic domain remains unaffected.
- *Degradation of the Insulator and Irreversible Charge Concentration*: When the micro-electrode is activated for a long duration, this causes irreversible charge concentration near the micro-electrodes. This results in an operation error and impedes the droplet motion. It can also lead to trapping of droplet in the micro-electrode. In the case of multiple micro-electrodes, this defect will lead to the splitting of the remaining portion of the droplet from the stuck droplet, hence further impeding the motion of the remaining droplet. Consequently, unintentional droplet operation would occur due to the variation in interfacial surface tension. This defect has an adverse effect on the droplet motion and its volume.
- *Short-Circuited Micro-Electrodes*: This happens due to etching defects. A metal connection between two micro-electrodes is formed by merging two micro-electrodes to form a single micro-electrode. Then, the droplet portion residing on this long micro-electrode is unable to move. Thus, the actuation of this portion of droplet is not achieved. This results in impeded motion and droplet volume reduction. This kind of defect can be found in the fluidic domain of the biochip.
- *Micro-Electrode Open Fault*: As each micro-electrode is connected to a separate activation circuitry beneath it, this fault leads to a non-activated micro-electrode when there is an opening in the metal connection between the micro-electrode and the activation circuitry. The droplet on the micro-electrode will not be able to move, causing variations in droplet shape, size and volume. This kind of defect originates from the electronic domain which would affect the fluidic domain.
- *Non-Uniform Dielectric Layer*: This is a manufacturing defect. This happens due to the coating failure on micro-electrodes. Due to this defect, the fragments of micro-droplets formed on the surface of micro-electrode could cause inaccurate volume of droplet being travelled to the destination. This defect exists in the fluidic domain.

Apart from the abovementioned defects, several bio-assay dependent defects such as protein fouling would also lead to erroneous behavior of biochips as the surface becomes permanently hydrophilic [76]. This fault is nearly unpredictable as it occurs during the bio-assay execution. We have observed that most of the defects that occur in the electronic domain would also affect the fluidic domain of MEDA-based digital microfluidics biochips. The defects in the fluidic domain are unique and independent from the activation circuitry underneath [10]. Thus, the testing of MEDA-based biochips becomes challenging if there exists some defects in the electronic domain which is not considered in the conventional DMFBs.

Recently, several works on synthesis and testing of MEDA-based microfluidics biochips have been presented [75,76,77,78,79,80,81]. A MEDA architecture-based bio-processor was proposed by Lai et al. [79] which was capable of performing extra functions such as self-testing, qualitative detector, data register and sensing results. This work was mainly related to the functioning of processor for various droplet activities such as merging, cutting and detection. The testing module in this work broadly focused on whether the defect was present or absent in the system regardless of the type of defect and the fault model. Later, a programmable lab-on-CMOS (LoCMOS) with micro-electrode cell array was presented by Lai et al. [78]. Operations such as droplet moving/cutting/mixing on a 2D micro-electrode array were demonstrated in this work. Also, an EDMOS high voltage switch was used to control and detect circuits in order to increase the avalanche voltage breakdown. Another synthesis approach for MEDA-based biochips was proposed by Li et al. [9] in which the scheduling, module placement, routing of droplet of various sizes were discussed. A general analytical model for droplet velocity is presented and validated through fabricated MEDA- based biochips. A size-aware droplet router was also proposed which is capable of routing the droplets of different sizes in MEDA-based biochips.

However, in order to obtain a high yield of MEDA-based biochips, testing of the MEDA architecture is needed before it is used for bio-assay operations. Limited amount of work has been done in the field of MEDA-based biochip testing [75]. Testing requirements for MEDA-based biochips was first discussed by Shukla et al. [75]. The advantage of diagonal movement of droplet in MEDA-based biochips for testing was also discussed by Shukla et al. [77]. In this approach, the capability of diagonal testing in detecting the undetectable faults was analyzed in comparison to parallel testing. Later, an oscillation-based testing methodology for the defect detection in MEDA-based biochips was presented by Shukla et al. [76] focusing on the offline error detection after the manufacturing process. The effects of change in droplet capacitances of single and multiple micro-electrodes on the oscillation-based sensing circuit were also analyzed. The results showed that any defect in micro-electrode would affect the output frequency. Recently, an efficient error recovery technique for on-line errors (errors produced during field operation of mixing/splitting) was proposed by Li et al. [80] for the MEDA-based digital microfluidics biochips. Local recovery methods based on Probabilistic–Timed Automata (PTA) were presented for error-recovery in MEDA-based biochips. Error recovery methods for localized error (e.g., unbalanced splitting and inadequate mixing) and complete bioassay have been discussed in this work. The vast potential of MEDA-based biochips in bio-medical applications can only be fully exploited when these biochips are reliable. Due to the limited work in the field of testing, there is a requirement of more testing approaches focusing on MEDA-based biochips. 

## 5. Conclusions and Future Work

Due to the complex architecture of MEDA, the number of testing work performed in this area is rather limited. Owing to various advantages of MEDA-based biochips over conventional DMFBs, its full potential must be explored and analyzed. This paper has presented various testing methodologies used for conventional digital microfluidic biochips. Testing methodologies such as structural testing, functional testing, error recovery, design for testability, etc. have pathed ways for DMFBs to be applied in various biomedical applications. Due to the increase in bioassay applications, MEDA-based biochips are more suitable in these bio-medical environments. Thus, the testing of these biochips is important. This paper has presented some previous works which are related to conventional DMFBs testing and MEDA-based biochip testing. Fault models for MEDA are also presented by focusing on types of defects occurring in these biochips. Thus, it is indeed necessary to develop new testing methodologies for MEDA-based biochips.

## Figures and Tables

**Table 1:** Summary of testing techniques for DMFBs.

Defect Type	Fault Model	Occurance of Defect	Testing Techniques Used	Related Work
Dielectric Breakdown	Droplet-electrode short	Manufcaturing/Online	(1) Structral testing (2) Defect tolerance (3) Error recovery methods	Su et al. [23,43] Mitra et al. [25]
Irreversible charge concentration	Electrode-stuck--on	Online	(1) Structral testing, (2) Defect tolerance, (3) Error recovery methods	Roy et al. [30]
Deformation of electrodes during fabrication	No overlap between mixing droplets and the center electrode	Fabrication defect	Functional testing	Xu et al. [37]
Particle contamination that connects two electrodes	Electrode-short	Manufacturing/Online	(1) Structural testing (2) Contamination-aware test methods	Roy et al. [68]
Sample residue on the surface of electrodes	Resistive open/contamination	Online	Cross-contamination testing methods	Zhao et al. [50,66]
Broken wire to control source due to abnormal metal layer	Electrode-open	Fabrication	(1) Structural testing (2) Error recovery	Hu et al. [54]
Unequal actuation voltages	Pressure gradient	Online	Functional testing	Xu et al. [38] Mitra et al. [39]
Non-uniform dielectric layer	Dielectric islands	Manufacturing/Online	Structural testing	Su et al. [43]

**Table 2:** Fault models for MEDA based biochips

Cause of Defect	Defect Type	Fault Model	Observable Error		Affected Domain
			N = 1	N > 1	
Excessive actuation of voltage applied to the micro-electrode	Dielectric breakdown	Droplet electrode short	Electrolysis of droplet, no further transportation	Dragged transportation of the remaining droplet	Fluidic domain
Micro-electrode actuation for long duration	Non uniform dielectric layer	Dielectric islands	Fragments of micro-droplets and its motion is prevented	Droplet shape is affected and their movement is prevented	Fluidic domain
Particle contamination	A particle that connects two or more adjacent micro--electrodes	Micro-electrode short	Droplet resides in one or more micro-electrodes	Unintentional shape and motion of droplet.	Fluidic domain
Excessive mechanical force applied to the biochip	Misalignment of parallel plates	Net static pressure in some direction	Droplet transportation without activation.	Transportation of droplet without activation	Fluidic + electronic domain
Protein adsorption during bioassay	(1) Grounding failure (2) Broken wire to control source (3) Metal connection between two adjacent micro-electrodes	(1) Floating fragments of droplets (2) Electrode open (electrode actuation not possible) (3) Short between two micro-electrodes	droplet sits in the middle of two micro-electrodes and transportation along one or more directions is not possible	Droplet shape, size and volume is affected and the droplet is unable to move	Fluidic domain
